# Treatment outcomes in symptomatic *Dientamoeba fragilis* infection: a prospective clinical and molecular study

**DOI:** 10.1007/s15010-026-02746-6

**Published:** 2026-02-23

**Authors:** Asaf Biber, Dafna Yahav, Eli Schwartz

**Affiliations:** 1 Infectious Diseases Unit, Sheba Medical Center, Ramat-Gan, Israel; 2 The Center for Travel and Tropical Medicine, Sheba Medical Center, Ramat Gan, Israel; 3 Faculty of Medical & Health Sciences, Tel-Aviv University, Ramat-Aviv, Tel-Aviv, Israel

**Keywords:** Gastrointestinal symptoms, Paromomycin, Metronidazole, Tinidazole, Albendazole, Antiprotozoal therapy, Molecular diagnosis, PCR, Clinical cure, D. fragilis pathogenicity

## Abstract

**Purpose:**

The clinical significance and optimal treatment of *Dientamoeba fragilis* (DF) infection remain controversial, despite its frequent detection in patients with chronic gastrointestinal symptoms. This study aimed to evaluate clinical and molecular responses to various antiparasitic regimens and to assess the association between symptom resolution and parasite eradication.

**Methods:**

In this prospective, single-center observational study conducted between January 2019 and June 2023, we included 105 symptomatic patients with a positive stool PCR for DF. Patients were treated with paromomycin (PAR), nitroimidazole monotherapy (NM; metronidazole/tinidazole), or a combination of tinidazole and albendazole (T + A). Clinical and molecular outcomes were assessed one month post-treatment using a structured symptom questionnaire and repeat PCR testing.

**Results:**

Of 96 patients with follow-up, 73 underwent repeat PCR testing. PAR was associated with significantly higher clinical and molecular cure rates (82.8% and 88.5%, respectively) compared to T + A (35.0% and 29.2%) and NM (4.2% and 10.0%). Combination therapy with T + A was superior to NM in clinical response but not in molecular clearance. A strong correlation was observed between clinical cure and DF eradication (p < 0.00001), reinforcing the pathogenic role of DF. Median symptom duration before referral was 9 months, indicating the chronic nature of untreated infection.

**Conclusions:**

DF should be considered in patients with unexplained chronic gastrointestinal symptoms. Molecular testing is recommended for accurate diagnosis. Paromomycin appears to be the most effective treatment, with a strong association between clinical recovery and microbiological cure. Randomized controlled trials are warranted to further define optimal management strategies for DF infection.

## Introduction

*Dientamoeba fragilis* (DF) is a binucleated flagellated protozoan and one of the most commonly detected intestinal parasites worldwide. Over the past decade, the use of molecular diagnostic techniques for stool testing has increased in many countries, particularly in the evaluation of gastrointestinal (GI) symptoms. As a result, DF has gained increased recognition and is now often referred to as an emerging protozoan pathogen [1].

Despite its high global prevalence, the pathogenic potential of DF remains controversial. When Dobell and Jepps first described the organism in 1918, they concluded it was nonpathogenic—even though six of the seven patients in their report presented with diarrhea or dysentery [2]. Several subsequent observations supported this nonpathogenic view, citing DF’s lack of tissue invasion and its frequent detection in asymptomatic individuals [3, 4].

A recent systematic review examining the causal role of DF in diarrheal illness reported conflicting results [5]. However,, DF infection may present with a broader spectrum of manifestations beyond diarrhea, including altered bowel habits, abdominal pain, bloating, and even extraintestinal features such as fatigue and eosinophilia [6].

In light of this uncertainty, treatment is still recommended for symptomatic patients in whom DF is the only identified pathogen. This recommendation is supported by the U.S. Centers for Disease Control and Prevention (CDC) and clinical platforms such as UpToDate and Medscape [7]. However, there is no consensus regarding the optimal treatment regimen. Metronidazole, historically considered first-line therapy [8], failed to demonstrate superiority over placebo in a randomized controlled trial involving children [9]. Observational studies have reported higher rates of clinical and microbiological cure with other agents, such as paromomycin, clioquinol, and ornidazole, compared to metronidazole [10–13].

Given these controversies, we conducted an observational study to assess the clinical and molecular response to various antiprotozoal agents commonly used in the treatment of symptomatic DF infection in routine clinical practice.

## Methods

A single-center, prospective observational study conducted at Sheba Medical Center between January 2019 and June 2023. We included consecutive patients presenting with chronic gastrointestinal (GI) symptoms lasting longer than one month and a positive stool PCR test for *Dientamoeba fragilis*, either as a monoinfection or co-infection with *Blastocystis* spp., Patients with elevated inflammatory markers were excluded.

Molecular testing was performed using the Allplex™ GI Parasite Assay (Seegene, South Korea), a one-step real-time PCR that detects gastrointestinal parasites including: *Blastocystis hominis*, *Cryptosporidium* spp., *Cyclospora cayetanensis*, *Dientamoeba fragilis*, *Entamoeba histolytica*, and *Giardia lamblia*.

At the initial visit, all patients completed a structured questionnaire that included travel history (when relevant), type and duration of symptoms, and the presence of the following gastrointestinal symptoms: loose stools, abdominal pain, bloating, flatulence and borborygmus, loss of appetite, weight loss, and fatigue (see Supplemental Appendix for questionnaire). Relevant prior investigations such as imaging studies or endoscopic procedures were also documented.

Treatment regimens were determined at the discretion of the attending physician or primary care provider. Some patients were prescribed a combination of tinidazole and albendazole, based on our clinic’s policy and previous experience with other intestinal protozoal infections [14]. Patients were asked to repeat the stool PCR test approximately one month after completing treatment.

Clinical cure was assessed one month post-treatment and defined as resolution or significant improvement in gastrointestinal symptoms, based on a yes/no response to the question:

“In regard to all your gastrointestinal symptoms, as compared with the way you felt before you started the study medication, have you had adequate relief of your gastrointestinal symptoms?” [15].

Molecular cure was defined as a negative stool PCR for DF at least one month following completion of treatment.

Patients who did not achieve clinical cure and remained PCR-positive were prescribed an alternative antiprotozoal therapy. Each treatment course, including multiple courses in the same patient, was evaluated independently. All patients with both clinical and molecular follow-up were included in the efficacy analysis. Patients without follow-up PCR were included in the symptom analysis. The first treatment course was assessed for clinical and molecular response, while all treatment courses were analyzed for correlation between clinical outcome and molecular clearance.

Statistical analysis was performed using Microsoft Excel 2016 (Microsoft Corp., Redmond, WA, USA). Continuous variables are presented as mean ± standard deviation (SD) or median with interquartile range (IQR). Categorical variables are reported as frequency and percentage. Group comparisons were performed using the Chi-square test or t-test for categorical and continuous variables, respectively. For expected frequencies < 5, Fisher’s exact test was used. Binary logistic regression was used to calculate odds ratios (ORs) and 95% confidence intervals (CIs) for clinical and molecular cure associated with each treatment. A p-value < 0.05 was considered statistically significant.

The study was approved by the Institutional Review Board (IRB) of Sheba Medical Center.

## Results

Over the four-and-a-half-year study period, 105 symptomatic patients were referred to our clinic with a positive PCR result for DF, either as monoinfection (n = 89, 84.8%) or in coinfection with *Blastocystis* spp. (n = 16, 15.2%). Demographic and clinical characteristics are summarized in Table 1.

**Table 1:** Demographic and Clinical Characteristics of the Study Population (N = 105)

Characteristic	Value
*Demographics*	
Age, years (median, IQR)	34 (21–49)
Female sex, n (%)	57 (54.3%)
Travel history prior to symptom onset, n (%)	47 (44.8%)
*Symptoms*	
Duration of symptoms, months (median, IQR)	9 (4–36)
Abdominal pain, n (%)	81 (77.1%)
Loose stools, n (%)	81 (77.1%)
Bloating, n (%)	56 (53.3%)
Fatigue, n (%)	58 (55.2%)
Weight loss, n (%)	29 (27.6%)

*Values are presented as n (%) or median (interquartile range, IQR)*

Patient ages ranged from 2.5 to 83 years, with a median age of 34 years. Seventeen patients (16.5%) were children under 18 years of age (median age 9 years), while the median age among adults (≥ 18 years) was 39 years. Females comprised 54.3% of the cohort (n = 57).

Forty-seven patients (44.8%) reported international travel prior to symptom onset. Travel destinations included the Indian subcontinent (n = 17), East and/or Southeast Asia (*n* = 8), Latin America (*n*= 7), Sub-Saharan Africa (*n* = 6), North Africa (*n*= 4), North America (*n*= 2), Central Europe (*n* = 2), and Eastern Europe (*n* = 1).

The most frequently reported symptoms were loose stools and abdominal pain, each occurring in 76.7% of patients, with a mean symptom duration of 9 months prior to referral (ranged 1 to 240 months). Seventeen patients (16.2%) underwent gastrointestinal endoscopy before their initial clinic visit, and all examinations were reported as normal.

Of the 105 patients, 96 (93.2%) returned for clinical follow-up, and 73 of these repeated also stool PCR testing.

Regarding treatment, 24 patients were prescribed nitroimidazole monotherapy (NM), either metronidazole 500 mg three times daily for 7–10 days or tinidazole 1 g twice daily for 2–3 days. Forty patients received a combination of tinidazole and albendazole (T + A), consisting of tinidazole 1 g twice daily for 2 days followed by albendazole 400 mg twice daily for 5 days. Twenty-nine patients were treated with paromomycin (PAR) 500 mg twice daily for 7 days. One patient received diloxanide, and two were treated with albendazole monotherapy.

Clinical and molecular cure rates following the first treatment course, stratified by treatment regimen, are presented in Table 2. Patients treated with PAR achieved a clinical cure rate of 82.8% (24/29) and a molecular cure rate of 88.5% (23/26), both significantly higher than those treated with T + A (clinical: 35% [14/40]; molecular: 29.2% [7/24]) or NM (clinical: 4.2% [1/24]; molecular: 10% [2/20]). T + A was significantly superior to NM in terms of clinical cure (p < 0.005), but not for molecular cure (Table 2).

**Table 2:** Clinical and Molecular Cure Rates According to Treatment Regimen

Treatment	N	Clinical Cure	OR⁽ᵃ⁾ (95% CI)	p-value	N (PCR)	Molecular Cure	OR⁽ᵃ⁾ (95% CI)	p-value
Paromomycin	29	82.8% (24/29)	110.4 (12.0–1018.5)	< 0.0001	26	88.5% (23/26)	69.0 (10.4–457.9)	< 0.0001
Tinidazole + Albendazole	40	35.0% (14/40)	12.4 (1.5–101.6)	< 0.0001	24	29.2% (7/24)	3.7 (0.7–20.4)	< 0.0001
Metronidazole/Tinidazole	24	4.2% (1/24)	Reference	Reference	20	10.0% (2/20)	Reference	Reference

*OR⁽ᵃ⁾* = *Odds Ratio for cure compared to metronidazole/tinidazole monotherapy (reference group)*

Eighteen patients who failed initial treatment with NM or T + A and remained PCR-positive for DF were subsequently treated with PAR. Among them, 72.2% (13/18) achieved clinical cure, and 82.4% (14/17) achieved molecular cure.

**Fig. 1:**
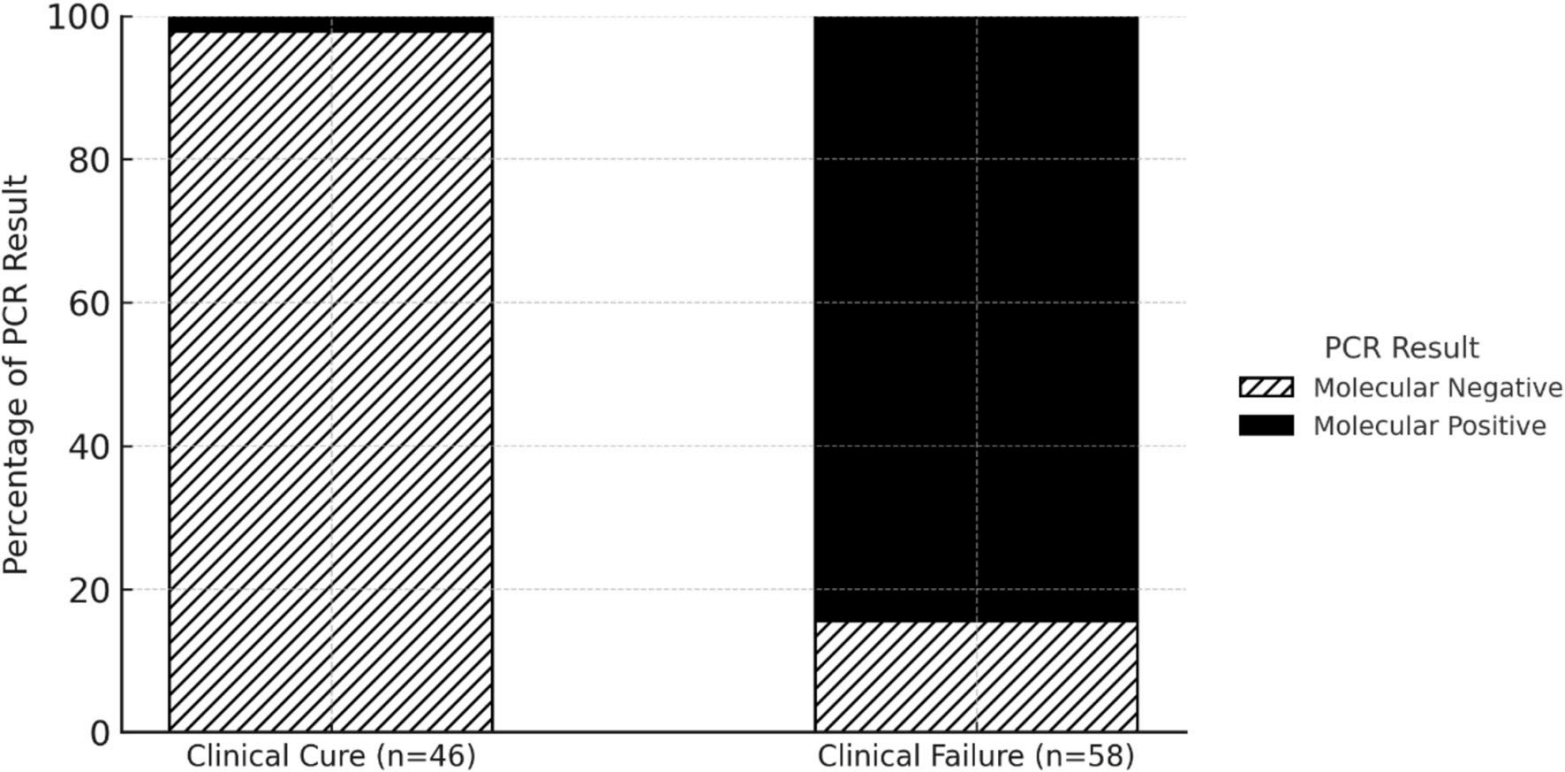
Molecular PCR result by clinical outcome. Proportion of molecular PCR results by clinical outcome. Patients with clinical cure showed a markedly higher proportion of PCR-negative results (98%) compared to those with clinical failure (15.5%)

Further, we analyzed the association between clinical and molecular cure. Among 104 treatment courses (NM, T + A, or PAR) with available data on both outcomes, clinical cure was observed in 46 cases, of which 45 (97.8%) also achieved molecular cure. In contrast, among 58 treatment courses resulting in clinical failure, 49 (84.5%) remained PCR-positive for DF (p < 0.00001; Fig. 1).

Patients with *Blastocystis* spp. coinfection were eligible for inclusion, as this organism is not considered pathogenic by most experts [16]. Coinfection with *Blastocystis* spp. was detected in 34 patients (33.0% of the cohort). Excluding these patients from analysis did not significantly alter the clinical or molecular cure rates (data not shown).

## Discussion

Historically, the clinical relevance of *Dientamoeba fragilis* (DF) has been difficult to establish, as diagnosis relied on microscopic examination of fresh stool specimens using special stains such as Giemsa or trichrome- a technically demanding approach given the fragile nature of the organism and the need for expert microscopists [2, 6].

However, DF is frequently detected when molecular diagnostic testing is used as part of the routine workup for persistent gastrointestinal symptoms [17]. Although PCR-based prevalence data should be interpreted with caution because of inter-assay variability and potential limitations in specificity[1, 6].

Despite ongoing debate regarding its pathogenicity, current clinical guidelines often recommend treatment in symptomatic patients with DF identified in the absence of other pathogens [18].

In our study, we observed significantly higher rates of both clinical and microbiological cure among symptomatic DF patients treated with paromomycin, compared to those treated with nitroimidazole-based therapy.

The optimal treatment for DF remains controversial. Metronidazole has traditionally been considered the standard of care, largely due to its in vitro activity against the parasite [10]. However, a randomized controlled trial conducted in children more than a decade ago demonstrated no clinical benefit of metronidazole over placebo [9]. Additionally, several retrospective studies have shown superior outcomes with paromomycin compared to metronidazole, consistent with our findings [12, 16, 19–21]. Although clinical response rates with metronidazole in previous studies were somewhat higher than in our cohort, paromomycin consistently outperformed it. The relatively poor performance of metronidazole in our study may reflect increasing resistance or reduced efficacy along the years.

Interestingly, our results also suggest that combination therapy with an imidazole and albendazole leads to improved clinical outcomes compared to imidazole monotherapy. Thus, this regimen may be considered when first-line alternatives such as paromomycin or clioquinol are not available.

A key finding in our study is the strong correlation between clinical cure and microbiological eradication of DF, as shown in Fig. 1. This association lends support to the assumption that DF is a true pathogen. Although DF has been known for over a century [2] uncertainty regarding its clinical relevance persists. Much of the debate centers on observational data indicating similar detection rates of DF in symptomatic and asymptomatic individuals [22]. However, our findings, together with those from a large Finnish cohort, challenge this assumption. In the Finnish study, involving 297 episodes of DF treated with paromomycin and 84 with metronidazole, microbiological cure was significantly associated with symptom resolution (OR 5.85 [95% CI: 3.02–11.32], p < 0.001) [16, 19].

Spontaneous clearance of DF has been described in previous studies [9, 12, 21], but our data highlight the chronic nature of symptomatic DF infections. The median symptom duration before presentation was nine months (IQR 4–36), suggesting that spontaneous resolution had not occurred and reflecting the hesitancy among clinicians to refer patients due to uncertainty regarding DF's pathogenic role.

Our study has several limitations. It was observational, non-randomized, and conducted in a single center in a single country. However, patients were referred from across the country, and our findings are consistent with reports from other regions, particularly Europe. Moreover, although not interventional, the study reflects real-world clinical practice, which may better capture treatment effectiveness in community settings. In addition, because the antiparasitic agents used have a relatively broad spectrum of activity, symptom resolution and parasite clearance cannot conclusively establish DF as the sole cause of the clinical presentation; however, the strong, consistent, and near-uniform correlation observed between molecular eradication of DF and clinical improvement strongly supports a pathogenic role for the organism.

Our study focused on parasitic agents only and did not include viral and bacterial pathogens since our study focused on patients with chronic gastrointestinal symptoms lasting longer than one month, and therefore the likelihood of an acute viral or bacterial etiology was considered low, as discussed previously [23].

In conclusion, DF should be considered in the differential diagnosis of patients with chronic gastrointestinal symptoms when no alternative etiology is found. Molecular testing is essential for accurate diagnosis. Our findings demonstrate a strong association between parasite eradication and symptom resolution, and paromomycin appears to be the most effective treatment currently available. However, randomized controlled trials are needed to validate these results and establish the optimal therapeutic approach for DF. Further studies are also required to clarify the pathogenicity and clinical importance of DF, including whether specific genotypes, subtypes, or strains are associated with symptomatic infection in humans.

## Data Availability

No datasets were generated or analysed during the current study.

## References

[1] Stark D, Al-Qassab SE, Barratt JLN, Stanley K, Roberts T, Marriott D, et al. Evaluation of multiplex tandem real-time PCR for detection of *Cryptosporidium* spp., *Dientamoeba fragilis*, *Entamoeba histolytica*, and *Giardia intestinalis* in clinical stool samples. J Clin Microbiol. 2011;49:257–62. 10.1128/JCM.01796-10. 21048004 10.1128/JCM.01796-10 PMC3020426

[2] Jepps MW, Dobell C. *Dientamoeba fragilis* n. g., n. sp., a new intestinal amoeba from man. Parasitology. 1918;10:352–67. 10.1017/S0031182000003929.

[3] Embree J. Dientamoeba fragilis: A harmless commensal or a mild pathogen? Paediatr Child Health. 1998;3:81–2. 10.1093/pch/3.2.81. 20401204 10.1093/pch/3.2.81 PMC2851273

[4] Jirků M, Kašparová A, Lhotská Z, Oborník M, Brožová K, Petrželková KJ, et al. A cross-sectional study on the occurrence of the intestinal protist, *Dientamoeba fragilis*, in the gut-healthy volunteers and their animals. Int J Mol Sci. 2022. 10.3390/ijms232315407. 36499734 10.3390/ijms232315407 PMC9737029

[5] Wong Z-W, Faulder K, Robinson JL. Does *Dientamoeba fragilis* cause diarrhea? A systematic review. Parasitol Res. 2018;117:971–80. 10.1007/s00436-018-5771-4. 29404747 10.1007/s00436-018-5771-4

[6] Stark D, Barratt J, Chan D, Ellis JT. *Dientamoeba fragilis*, the neglected trichomonad of the human bowel. Clin Microbiol Rev. 2016;29:553–80. 10.1128/CMR.00076-15. 27170141 10.1128/CMR.00076-15 PMC4861990

[7] https://www.cdc.gov/parasites/dientamoeba/health_professionals/index.html.

[8] Nagata N, Marriott D, Harkness J, Ellis JT, Stark D. In vitro susceptibility testing of *Dientamoeba fragilis*. Antimicrob Agents Chemother. 2012;56:487–94. 10.1128/AAC.05125-11. 22024820 10.1128/AAC.05125-11 PMC3256011

[9] Röser D, Simonsen J, Stensvold CR, Olsen KEP, Bytzer P, Nielsen HV, et al. Metronidazole therapy for treating dientamoebiasis in children is not associated with better clinical outcomes: a randomized, double-blinded and placebo-controlled clinical trial. Clin Infect Dis. 2014;58:1692–9. 10.1093/cid/ciu188. 24647023 10.1093/cid/ciu188

[10] Schure JMAT, de Vries M, Weel JFL, van Roon EN, Faber TE. Symptoms and treatment of *Dientamoeba fragilis* infection in children, a retrospective study. Pediatr Infect Dis J. 2013;32:e148–50. 10.1097/INF.0b013e31827f4c20. 23190787 10.1097/INF.0b013e31827f4c20

[11] Hazenberg HMJL, Mank TG, Band C, Euser SM, van Soest EJ. A prospective analysis of clinical and parasitological outcomes after treatment or a wait-and-see approach of Dientamoeba fragilis infection in an adult general practice population. Eur J Clin Microbiol Infect Dis. 2025;44:143–50. 10.1007/s10096-024-04989-3. 39540993 10.1007/s10096-024-04989-3

[12] Burgaña A, Abellana R, Yordanov SZ, Kazan R, Pérez Ortiz AM, Ramos CC, et al. Paromomycin is superior to metronidazole in Dientamoeba fragilis treatment. Int J Parasitol Drugs Drug Resist. 2019;11:95–100. 10.1016/j.ijpddr.2019.10.005. 31759244 10.1016/j.ijpddr.2019.10.005 PMC6880088

[13] Kurt O, Girginkardeşler N, Balcioğlu IC, Ozbilgin A, Ok UZ. A comparison of metronidazole and single-dose ornidazole for the treatment of dientamoebiasis. Clin Microbiol Infect. 2008;14:601–4. 10.1111/j.1469-0691.2008.02002.x. 18397330 10.1111/j.1469-0691.2008.02002.x

[14] Meltzer E, Lachish T, Schwartz E. Treatment of giardiasis after nonresponse to nitroimidazole. Emerg Infect Dis. 2014;20:1742–4. 10.3201/eid2010.140073. 25271363 10.3201/eid2010.140073 PMC4193167

[15] Krause R, Zollner-Schwetz I, Valentin T. Rifaximin for Irritable Bowel Syndrome without Constipation. N Engl J Med. 2011;364:1467–8. 10.1056/NEJMc1101839. 21488771 10.1056/NEJMc1101839

[16] Menéndez C, Fernández-Suarez J, Boga Ribeiro JA, Rodríguez-Pérez M, Vázquez F, Gonzalez-Sotorrios N, et al. Epidemiological and clinical characteristics of *Dientamoeba fragilis* infection. Enferm Infecc Microbiol Clin. 2019;37:290–5. 10.1016/j.eimc.2018.07.008. 10.1016/j.eimc.2018.07.008 30274823

[17] Peretz A, Azrad M, Ken- Dror S, Strauss M, Sagas D, Parizada M, et al. The epidemiology of intestinal protozoa in the Israeli population based on molecular stool test: a nationwide study. Microbiol Spectr. 2024. 10.1128/spectrum.00616-24. 39012121 10.1128/spectrum.00616-24 PMC11302726

[18] van Gestel RS, Kusters JG, Monkelbaan JF. A clinical guideline on Dientamoeba fragilis infections. Parasitology. 2019;146:1131–9. 10.1017/S0031182018001385. 30165915 10.1017/S0031182018001385

[19] Pietilä J-P, Häkkinen TA, Pakarinen L, Ollgren J, Kantele A. Treatment of *Dientamoeba fragilis*: a retrospective Finnish analysis of faecal clearance and clinical cure comparing four antiprotozoal drugs. New Microbes New Infect. 2023;54:101179. 10.1016/j.nmni.2023.101179. 37786407 10.1016/j.nmni.2023.101179 PMC10542007

[20] Clemente L, Pasut M, Carlet R, Ruscio M, Fontana F. *Dientamoeba fragilis* in the North-East of Italy: prevalence study and treatment. Parasitol Int. 2021;80:102227. 10.1016/j.parint.2020.102227. 33137500 10.1016/j.parint.2020.102227

[21] van Hellemond JJ, Molhoek N, Koelewijn R, Wismans PJ, van Genderen PJJ. Is paromomycin the drug of choice for eradication of *Dientamoeba fragilis* in adults? Int J Parasitol Drugs Drug Resist. 2012;2:162–5. 10.1016/j.ijpddr.2012.03.002. 24533277 10.1016/j.ijpddr.2012.03.002 PMC3862430

[22] Holtman GA, Kranenberg JJ, Blanker MH, Ott A, van Lisman- Leeuwen Y, Berger MY. *Dientamoeba fragilis* colonization is not associated with gastrointestinal symptoms in children at primary care level. Fam Pract. 2017;34:25–9. 10.1093/fampra/cmw111. 27784723 10.1093/fampra/cmw111

[23] Schwartz E, Connor BA. Persistent abdominal symptoms: a persistently neglected topic in travel medicine. J Travel Med. 2022. 10.1093/jtm/taac016. 35294014 10.1093/jtm/taac016

